# Ancient frameworks as modern templates: exploring reef rubble consolidation in an ancient reef system

**DOI:** 10.1098/rspb.2024.2123

**Published:** 2025-02-05

**Authors:** Amanda Godbold, Chase C. James, Wolfgang Kiessling, Niklas Hohmann, Emilia Jarochowska, Frank A. Corsetti, David J. Bottjer

**Affiliations:** ^1^ Department of Earth Science, University of Southern California, Los Angeles, CA, USA; ^2^ Department of Geography and Geosciences, Friedrich-Alexander University Erlangen-Nuremberg, Erlangen, Germany; ^3^ Department of Earth Sciences, Utrecht University, Utrecht, The Netherlands

**Keywords:** Late Triassic, reef, reef rubble, reef regeneration, breccia

## Abstract

Both natural and human-induced stressors cause reef erosion, resulting in reef rubble formation. When consolidated, the rubble can facilitate reef recovery, sparking interest in artificial rubble stabilization as a method for reef restoration. However, our understanding of the natural processes governing coral reef regeneration within rubble beds is limited. This study examines the regeneration processes within ancient rubble frameworks in a Late Triassic carbonate platform. Results show that Late Triassic rubble environments exhibit successional trajectories similar to contemporary rubble environments. Key organisms such as sponges, calcareous red algae, bryozoans, microbes and scleractinian corals, which are instrumental in the consolidation of modern reef rubble, appear to have played comparable roles during the Late Triassic. The similarities between Late Triassic and modern reef rubble consolidation highlight enduring ecological mechanisms important for reef regeneration. This study deepens our understanding of reef dynamics and offers valuable insights for improving current reef restoration strategies, grounded in time-tested natural processes.

## 1. Introduction

Although the ecology of living reefs has been extensively studied, the ecological dynamics of reef rubble—especially its importance in optimizing coral reef restoration—have only recently begun to receive significant attention [1–3]. Investigating reef rubble from ancient reef systems presents a valuable opportunity to examine this ecosystem’s dynamics through their cycles of destruction and regeneration over timescales not observable in recent environments.

Coral rubble encompasses a wide range of sizes, from pieces larger than sand to boulder-sized fragments [1]. It forms when mechanical, biological or chemical processes break apart live coral colonies or reef structures, producing coral fragments or reef rock rubble. Rubble generation is an inherent component of reef ecosystems, with reef cores typically accumulating layers of unconsolidated and consolidated rubble over millennia [1,4–7]. However, unconsolidated rubble can create a hostile environment for many primary reef-builders, as the movement of rubble can hinder the survival of new recruits [1–3,8]. Consequently, reef regeneration following physical disturbance requires initial substrate stabilization, which may then be followed by rubble binding and consolidation. The binding and consolidation process generally involves two stages: an initial binding phase that provides temporary stability, and a subsequent, more permanent phase of rigid binding and lithification [1,8,9].

Preliminary stabilization ensures that rubble remains temporarily stable under average wave conditions. This is achieved in low hydrodynamic energy environments through the interlocking of rubble pieces, the presence of seagrass and/or colonization by pioneering organisms [1,8,9]. If it remains stable, modern reef rubble can be bound by soft binders like macroalgae and sponges, and over time, rubble achieves permanent rigidity through marine cementation and/or the lateral growth of calcareous organisms, with crustose coralline algae being particularly effective in this binding process [1,8]. Additionally, recent research has highlighted the significant role of microbes in enhancing binding through microbially driven lithification within these environments [10,11].

Despite continued efforts to understand the role of rubble consolidation in reef regeneration, significant gaps persist in our knowledge of the natural processes that drive rubble stabilization, consolidation and reef recovery. For instance, the factors contributing to rubble formation, the natural mechanisms of coral recovery in rubble-dominated areas and the critical threshold beyond which rubble fields may no longer revert to their original ecological states without intervention are still not fully understood. Palaeontological data, which span thousands of years and include multiple physical disturbances, offer valuable insights into these processes [12]. This includes the exploration of critical thresholds by examining cases where reefs successfully recovered versus those where recovery failed, and comparing the ecological assemblages in both scenarios.

This study investigated the stages of reef regeneration within rubble fields located along the Upper Triassic Dachstein platform (*ca* 220 million years ago), now part of the Northern Calcareous Alps in Austria. The Triassic Period marked a critical evolutionary juncture in reef ecosystems, characterized by the advent of the first scleractinian coral reefs and the widespread proliferation of elaborate reef structures predominantly built by calcisponges and scleractinian corals [13–15]. We explored whether the fossil organisms in these Triassic reef rubble environments performed consolidating functions comparable to those of their modern counterparts.

We analysed the taxonomic composition of various stages of reef regeneration to clarify the structural and ecological roles of these organisms throughout the reef recovery process. Our study aimed to determine whether Triassic organisms performed analogous structural and ecological functions to their modern counterparts in rubble environments by examining their relative abundances, general morphologies and encrusting relationships (i.e. the patterns of encrustation sequences, including the typical taxa involved and the order in which different encrusting organisms colonize a substrate).

## 2. Methods

### (a) Field methods

Data were collected from three outcrops: one outcrop from Feisterscharte (outcrop 1: 47°27′03.0″ N 13°41′26.0″ E) and two from Gosaukamm (outcrop 2: 47°30′59.88″ N 13°29′25.422″ E; outcrop 3: 47°30′52.2″ N 13°29′17.8″ E). During the Late Triassic, reefs in this area developed within a sheltered shallow marine setting that was periodically disrupted by high-energy events, likely tropical storms or hurricanes. These disturbances resulted in significant deposits of reef breccia (i.e. reef rubble) that spread across the platform [16–18]. According to the conodont-based biostratigraphic framework for the Dachstein platform [19], all our samples belong to the Norian stage of the Late Triassic. The samples from Feisterscharte span the early to middle Lacian (early Norian, ~227.3–217.5 Ma), while samples collected from Gosaukamm cover the late Alaunian (middle Norian, ~217.5–214 Ma) to the late Sevatian (late Norian, ~214 to ~209–206 Ma) periods [20]. This temporal and geological framework offers a detailed context for exploring the long-term dynamics involved in reef regeneration and rubble consolidation.

**Figure 1.**
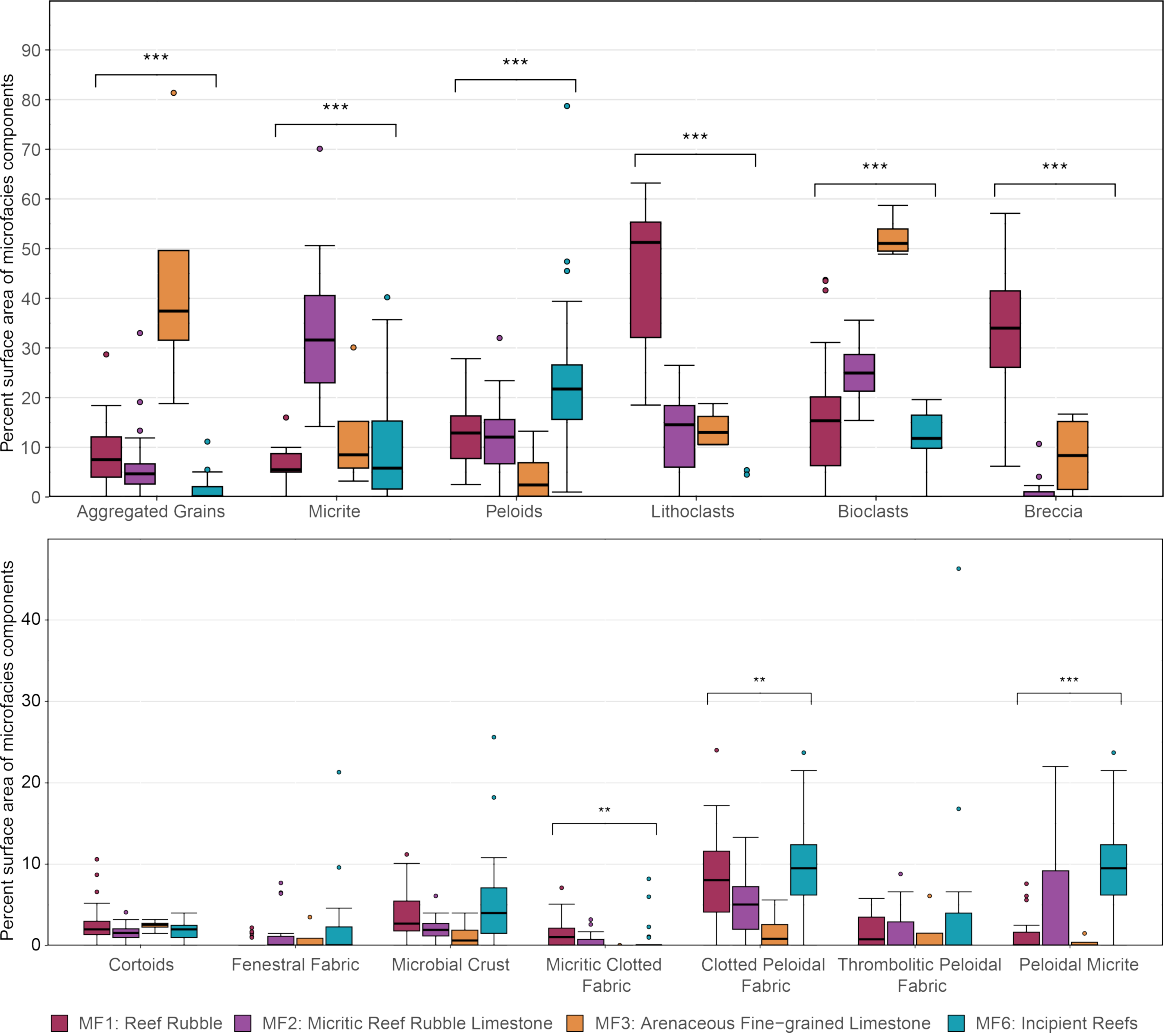
Average percent surface area of microfacies components. The upper panel shows the main components used to differentiate microfacies, while the lower panel highlights textures mediated by microbial activity. The total number of samples analysed is 73.

At Gosaukamm (outcrop 2) and Feisterscharte (outcrop 1), a 10 × 10 m^2^ quadrat, oriented along the geological strike, was randomly positioned for comprehensive field observations and sample collection. Due to outcrop restrictions, a 10 × 8 m^2^ quadrat was used at Gosaukamm (outcrop 1). The quadrats consisted of four to five vertically stacked 10-metre-long transects, oriented parallel to the strike and spaced 2 m apart. Each transect provides a representation of the spatial distribution of the data. Photographs were taken along the entire length of each transect, and samples were systematically collected at 2 m intervals. Within a quadrat, a vertical transect across multiple transects represents a progression through time, from older to younger layers as one moves upward along the quadrat (see figure 1). In contrast, the meterage along a single transect reflects spatial relationships that are time-equivalent. This systematic sampling method was designed to effectively capture both spatial and temporal variations in the dataset.

A total of 84 samples were collected across all quadrats, processed into 7 cm² thin sections oriented perpendicular to bedding, and photographed for microfacies analysis. A microfacies refers to a specific set of microscopic characteristics that helps to interpret its depositional environment and formation processes. Each sample was categorized into a microfacies type (MF) according to the classification system defined by Wurm [17] for the Gosaukamm Range. We adhered to Wurm’s nomenclature to ensure consistency and accuracy in the classification of each microfacies. Due to sample quality, 11 samples could not be processed into thin sections, resulting in 73 thin sections. High-resolution images of thin sections were stitched together with Zeiss ZEN 2.3 software to create single high-resolution mosaic images. The original orientation of each sample within the outcrop was marked on each thin section and corresponding image. Microfacies analysis, both quantitative and semi-quantitative, involved 88 identified taxa and 41 textural components.

Following the recommendations of Flügel & Munnecke [21] for coarse-grained limestones, an area-based approach using Adobe Illustrator was employed to gather fossil abundance data [21]. A 0.5 cm² grid was overlaid on the thin-section images, and each individual grid box was examined to obtain individual count data for calcisponges, scleractinian corals, calcareous algae, microproblematica, bryozoans, hydrozoans and encrusting foraminifera. The areas of textural components were also quantified within each grid box by outlining each component using the Adobe line tool and calculating the percent surface area. This process involved outlining each textural component using Adobe Illustrator’s drawing tools and measuring the occupied area to determine the percentage of surface area. For detailed information on the datasheet organization and access, see the electronic supplementary material. Growth forms, encrusting sequences, and spatial orientations observed in thin-section analyses were recorded to categorize each species into two categories: binding forms (irregular growth patterns connecting rubble or reef-building organisms) and erect forms (distinct, vertically expansive structures not observed as binders in thin-section analyses).

### (b) Statistical analysis

All analyses were performed in R software (R Core Team, 2024), version 4.3.2. Differences in the percent surface area of microfacies components between microfacies representing different stages of reef regeneration were visualized with a box–whisker plot (figure 1). For all statistical analyses, samples from the three outcrops were grouped into microfacies and analysed using the methods outlined below. The code, data and supplementary tables are available on the Open Science Framework repository (https://doi.org/10.17605/OSF.IO/GBECX). We utilized permutational multivariate analysis of variance (PERMANOVA) to examine the effects of microfacies and location on both textural components and taxonomic composition across sampled outcrops. Separate PERMANOVA analyses were conducted for textural and taxonomic data, employing a Bray–Curtis dissimilarity matrix with 999 permutations to assess the statistical significance of each factor.

To further dissect the contributions of textural elements and taxa to compositional differences among microfacies, we applied the Similarity Percentage (SIMPER) procedure. Key textural dissimilarities are detailed in §3.1, with comprehensive results available in electronic supplementary material, ‘simper_micro’. For taxonomic dissimilarities, we focus on comparisons between microfacies MF2 and MF6, as they feature *in situ* fossil taxa critical for subsequent ecological analyses. *In situ* fossil taxa are those preserved in their original growth position, indicating that they have not been transported or re-deposited after death.

A comprehensive table with results across all microfacies is available in electronic supplementary material, file ‘simper_taxa’. For this analysis, we prioritized taxa and textural components with high average contributions to dissimilarity and consistency across samples, as indicated by a high average-to-standard deviation ratio. Key taxa and textural components were defined as those cumulatively accounting for up to 70% of the observed dissimilarity between microfacies. An introduction to the SIMPER tables is included in the electronic supplementary material.

Spearman’s rank order correlation coefficient (**ρ**) assessed the strength and direction of monotonic relationships between species occurrence data, with results presented on a correlogram using the ‘corrplot’ function. To explore relationships among variables further, a probabilistic model [22] implemented with the ‘cooccur’ R package [23] uncovered associations between distinct taxonomic groups within the defined microfacies, using a presence–absence matrix. The null hypothesis in co-occurrence analysis is that the distribution of species is independent and random, with no ecological interactions driving the observed co-occurrence patterns. Results were displayed using a co-occurrence network constructed with the ‘cooccur’ and ‘visNetwork’ packages.

In a co-occurrence network, nodes represent individual taxa, and edges indicate the co-occurrence relationships between them. The ecological motivation for using co-occurrence network analysis resides in its ability to reveal patterns of species interactions and associations within an ecosystem [24]. By identifying which taxa frequently co-occur, potential ecological relationships can be inferred. To perform Spearman’s rank correlation and co-occurrence analyses, data were grouped by microfacies, combining all samples of the same microfacies across all outcrops. Only data from MF6 and MF2 were included, as these microfacies contain *in situ* fossil taxa, which is essential for assessing ecological relationships. This approach ensures that taxa originated from the same depositional setting and were not transported post-mortem, preserving the accuracy of ecological inferences.

Combining Spearman’s correlation with co-occurrence analysis provides a robust approach to understanding species interactions and distributions in ecological studies. Spearman’s correlation reveals the strength and direction of relationships between species’ abundances, while co-occurrence analysis identifies patterns of species presence or absence, indicating potential ecological relationships or shared habitat preferences. Together, these methods offer a more comprehensive understanding of species dynamics, enhancing the depth and reliability of ecological interpretations.

## 3. Results

### (a) Microfacies characteristics

We identify four microfacies types (MF1, MF2, MF3 and MF6 following [17]). PERMANOVA analyses suggest that 56.5% (*F* = 33.36, *p* = 0.01) of the total variance in textural components is explained by microfacies, demonstrating strong textural differentiation among microfacies types. Location accounted for 4.4% of the variation (*F* = 3.93, *p* = 0.01), signifying minor yet significant textural distinctions between locations. Additionally, the interaction between microfacies and location explained 4.6% of the variance (*F* = 1.37, *p* = 0.04), suggesting a modest but statistically significant interaction effect. The SIMPER analysis reveals that lithoclasts, bioclasts, brecciated cement and micrite are the primary contributors to dissimilarity between groups, with aggregated grains and interparticle cement also playing a notable, though lesser, role (electronic supplementary material, table ‘simper_micro’).

The reef rubble microfacies (MF1) is characterized by the dominance of angular lithoclasts (i.e. reef rubble) ranging in size from sand to boulder (1 mm to 7 cm) (grain classifications based on Wentworth–Udden grain-size scale). These lithoclasts exhibit a variety of textures typical of reef-derived clasts. MF1 is notable for its absence of *in situ* fossils or autochthonous textures, which are textures formed and preserved at their original site of deposition without significant alteration post-deposition. Brecciated cement and lithoclasts are the key identifying features of this microfacies (figure 1).

The micritic reef rubble microfacies (MF2) incorporates reef-derived materials in a microcrystalline calcite matrix, commonly known as ‘micrite’ or ‘carbonate mud’. Micrite is the distinctive feature of this microfacies (figure 1). The matrix can vary considerably within a single thin section, ranging from micrite to peloidal micrite. Like the incipient reef microfacies (MF6), MF2 contains hardgrounds suggestive of early lithification. Additionally, this microfacies includes both bioclastic material and *in situ* fossil taxa.

The arenaceous fine-grained microfacies (MF3) is characterized by an abundance of well-sorted, rounded skeletal fragments within a sparry calcite matrix. This microfacies exhibits the highest abundance of aggregated grains (figure 1). Similar to MF1, MF3 lacks *in situ* fossils and autochthonous textures.

The incipient reef microfacies (MF6) is characterized by the presence of reef-building organisms, predominantly calcisponges and, to a lesser extent, scleractinian corals, which are typically found *in situ* and are frequently encrusted by multiple layers of biogenic material. MF6 exhibits the highest proportion of *in situ* microbial textures (figure 1) and intra-interparticle cement—components conducive to early lithification. The consolidation of these incipient reefs likely occurred through extensive encrustation, primarily by microbial crusts and calcareous organisms, alongside rapid submarine cementation as evidenced by the abundance of hardgrounds. Thin-section images for samples belonging to each of the described microfacies can be found in electronic supplementary material, figure S1.

**Figure 2.**
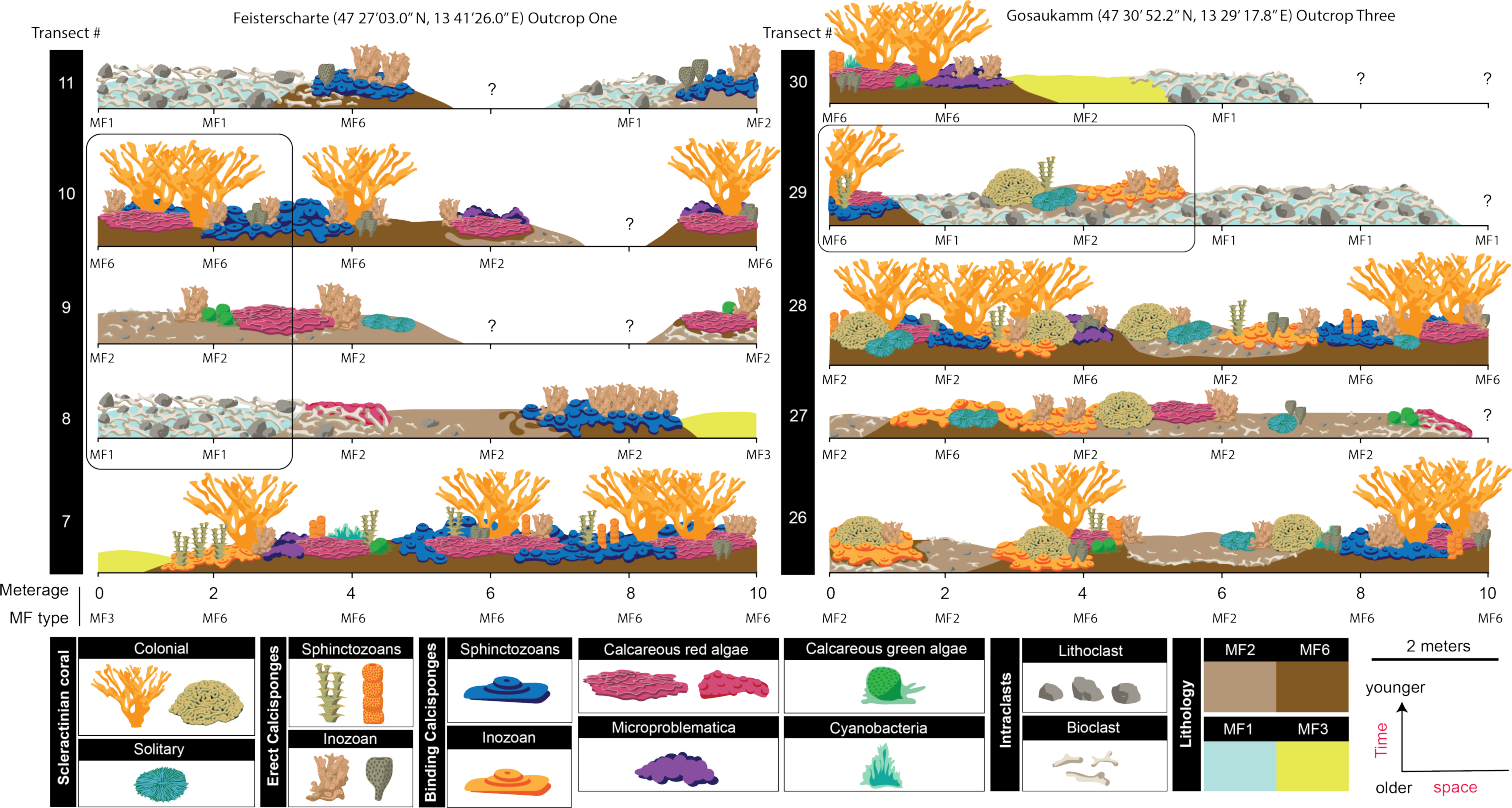
Reconstruction of the spatial and temporal arrangement of microfacies found at two of the studied outcrops. To maintain figure resolution within size constraints, only one Gosaukamm outcrop is included, illustrating 10 of the 14 transects: 5 from Feisterscharte (left) and 5 from Gosaukamm outcrop 2 (right). Within the illustration, each row corresponds to a transect. The spatial distribution of microfacies is shown along the *x*-axis, while the temporal distribution is depicted along the *y*-axis, with older transects positioned at the bottom and younger ones at the top. This illustration was created using data from thin sections, including abundance and microfacies components, to accurately reconstruct the composition along each transect. The question marks denote the samples that were not processed into thin sections. The two black boxes outline a temporal and spatial example of the different stages of reef regeneration discussed in the study.

All four microfacies are found at each outcrop and can be found along a single coeval transect, demonstrating their close spatial relationship (ranging from 2 to 10 m apart) at the time of deposition (figure 2). Furthermore, these microfacies exhibit a progression from primarily parautochthonous material in MF1 to predominantly autochthonous material in MF6, alongside an increase in the structural complexity of *in situ* fossil communities reflecting stages of reef regeneration (figure 2).

### (b) Taxonomic composition of microfacies

PERMANOVA indicates that microfacies account for 11.1% of the variation in taxonomic composition (*F* = 3.31, *p* = 0.01), with location contributing similarly at 11.0% (*F* = 1.37, *p* = 0.04), reflecting a moderate influence of both factors on community structure. The interaction between microfacies and location was also significant, explaining 9.8% of the variance (*F* = 1.46, *p* = 0.01), which implies that the effect of microfacies on taxonomic composition varies by location. Despite these distinctions, only a few taxa were unique to specific outcrops, indicating substantial overlap in community composition across outcrops. Outcrop 2 contained unique taxa such as the corals *Distichomeandra dieneri* and *Retiophyllia oppeli*, and benthic foraminifer *Glomospira* sp., while outcrop 3 was distinguished by taxa like Problematica 1, *Trochammina* sp. and *Turrispirillina* sp.

Since MF1 and MF3 are composed entirely of transported fossil fragments, our analysis concentrated on MF2 and MF6, which preserve *in situ* fossil assemblages.

SIMPER analysis revealed several key taxa driving the compositional differences between MF2 and MF6, collectively accounting for up to 70% of the observed dissimilarity. For detailed results, refer to table ‘simper_taxa’ in the electronic supplementary material. Among the most influential contributors to these differences were benthic foraminifera and specific calcisponges, which played prominent roles in shaping the distinct community compositions of these microfacies.

#### (i) Taxonomic composition of microfacies 2 and 6

**Figure 3.**
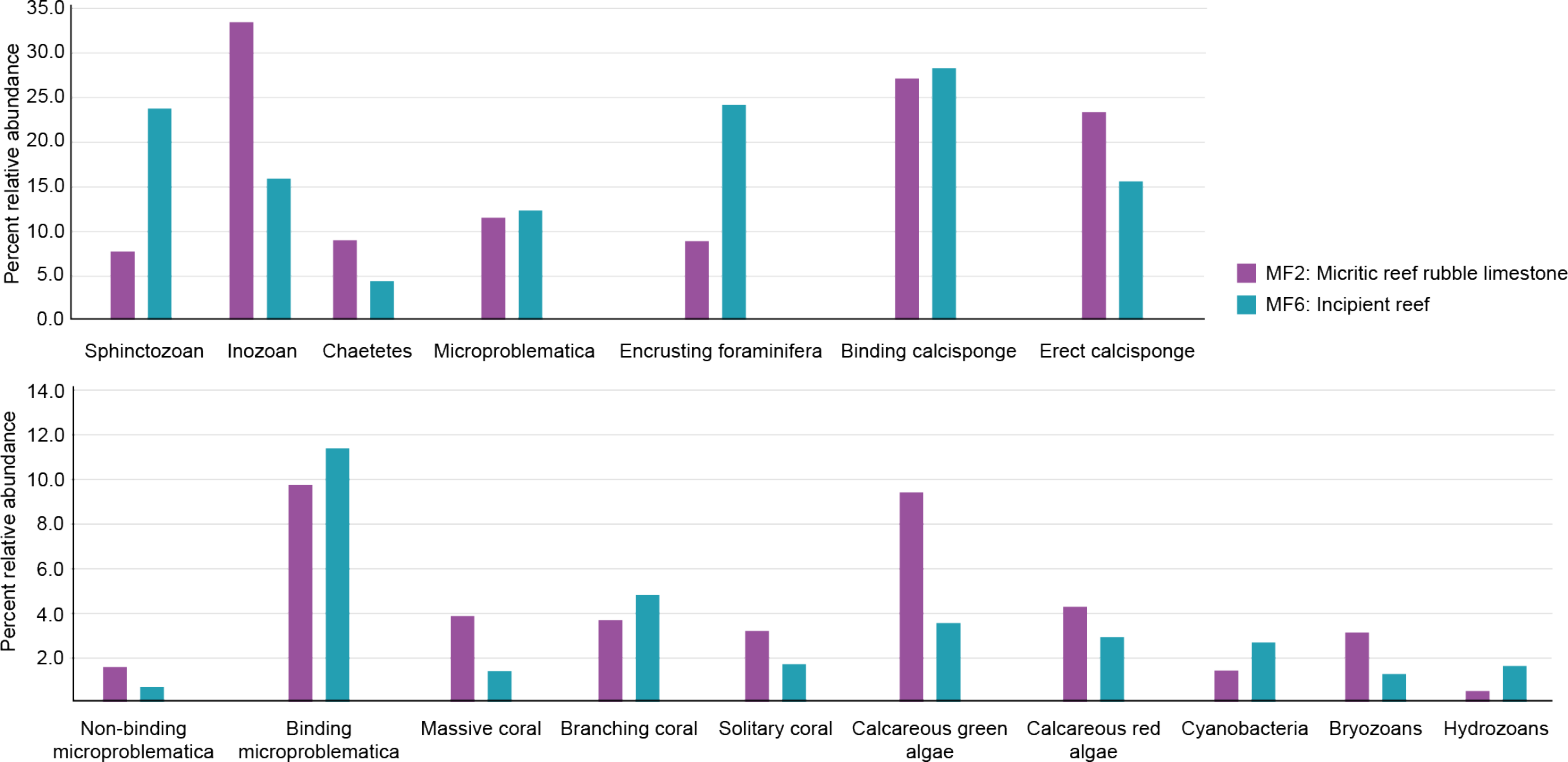
Taxonomic composition of the two major microfacies that contain *in situ* fossil taxa, MF2 and MF6. This bar graph shows the percent relative abundance of fossil taxa found within the two microfacies that contain *in situ* fossil taxa: incipient reef (MF6) and micritic reef rubble (MF2). The total number of samples across MF2 and MF6 = 43.

Calcisponges are the dominant organisms in the samples of MF2 and MF6, represented by 24 species classified into 15 binding species, 12 erect species and 1 boring ichnospecies. In this study, binding calcisponges are classified as secondary frame-building organisms, playing a crucial role in reinforcing reef structures. In contrast, erect calcisponges are identified as primary frame-building organisms, as they actively contribute to the foundational development of reefs by forming the core framework upon which the reef ecosystem is built. Within these microfacies, binding calcisponges have the highest occurrences (*n* = 577), followed by erect calcisponges (*n* = 441). Specifically, binding calcisponges account for a higher percentage of the taxonomic composition in MF6, whereas binding and erect calcisponges show similar percentages in MF2 (figure 3). Segmented calcisponges, known as sphinctozoans, have the highest occurrence in MF6 (*n* = 341) compared to MF2 (*n* = 58). Meanwhile, non-segmented calcisponges, called inozoans, exhibit comparable occurrences in both microfacies (MF2, *n* = 251; MF6, *n* = 229). Notably, inozoan calcisponges feature the highest relative abundance in MF2, while sphinctozoans have the highest relative abundance in MF6 (figure 3).

A total of 14 scleractinian coral species across 11 genera were identified and grouped into broad morphotypes, yielding 5 branching, 6 massive and 3 solitary species. Among these, branching corals, particularly from the genera *Cycliphyllia* and *Retiophyllia*, are the most common (*n* = 98), followed by solitary (*n* = 48) and massive corals (*n* = 48). Branching corals constitute the largest percentage of coral morphotypes found in MF6, while massive corals make up the largest percentage of MF2 (figure 3). Many branching corals in MF2 are found as fragments rather than intact colonies in their growth positions, resulting in massive and solitary corals comprising a larger proportion of the *in situ* coral assemblage (figure 3).

Conversely, within MF6, massive and solitary corals comprise notably smaller proportions of the coral assemblage. Branching corals significantly enhance the structural complexity of these reefs, followed by massive corals and, to a lesser extent, erect calcisponges.

Microbial fabrics are abundant in the analysed samples, constituting a considerable percentage of the surface area in each one (figure 3). These textures are commonly seen binding lithoclasts and reinforcing the bases of primary frame-building organisms like scleractinian corals and calcisponges.

Calcareous red algae, belonging to the genus *Parachaetetes*, make up a greater relative abundance in MF2 than in MF6 (figure 3). Thin-section analysis and outcrop observations indicate that these algae played a crucial role in binding reef-derived debris and fortifying the bases of primary framework-building organisms. Microproblematica, a term for microscopic fossil organisms with uncertain biological origins, accounted for a comparable proportion of the community in both microfacies (figure 3). Thin-section analysis revealed that microproblematica, similar to calcisponges and calcareous red algae, contributed to binding and fortifying reef structures. Green calcareous algae were more abundant in MF2 (figure 3). Encrusting foraminifera, calcareous cyanobacteria and hydrozoans were more prominent in MF6. These organisms are readily observed in thin-section encrusting primary framework-building organisms, thereby strengthening the overall reef structure. Lastly, bryozoans made up a larger proportion of the fossil community found in MF2 than in MF6 (figure 3).

### (c) Fossil relationships

**Figure 4.**
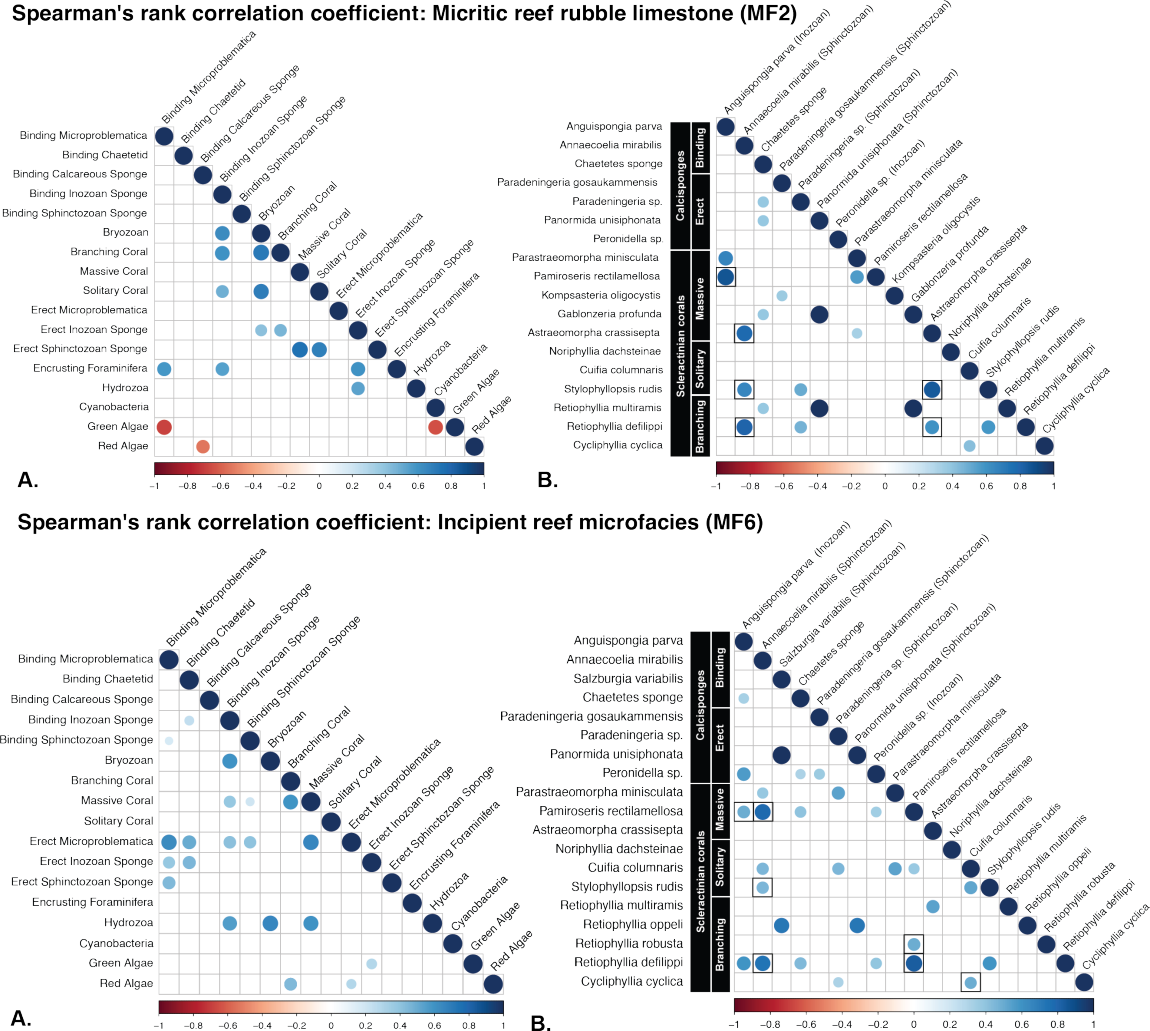
Spearman’s rank correlation analysis. Circles represent significant correlations (*p* < 0.05) between variables, while absence of a circle (blank cells) represents a non-significant correlation. In the correlogram, the size and colour of the circles represent the correlation coefficient (*R*), shown in the legend below (−1 to 1). A value of +1 indicates a perfect positive relationship, −1 a perfect negative relationship and values near 0 indicate little or no relationship. The circle sizes in the correlogram vary to reflect the strength of the correlation coefficient (*R*). Larger circles indicate stronger correlations (closer to +1 or −1), while smaller circles represent weaker correlations (closer to 0). The top panels display results from samples belonging to MF2 while the bottom panels display results from samples belonging to MF6. (A) Correlogram showing the most correlated variables (Spearman correlations) among raw abundance data for broad organism groups. (B) Correlogram showing the most correlated variables among raw abundance data for each coral species found and specific sponge species.

Within MF2, there are positive correlations between both binding and erect inozoan calcisponges and several primary frame-building organisms, such as branching scleractinian corals, as well as pioneering taxa like bryozoans and encrusting foraminifera (figure 4). In contrast, binding and erect sphinctozoan sponges show limited correlations.

Within MF6, most positive correlations involving inozoan calcisponges occur with secondary frame-building taxa such as other calcisponges, hydrozoa and microproblematica (figure 4). Again, binding and erect sphinctozoan sponges show limited correlations. In contrast to MF2, calcareous red algae exhibit a positive correlation with branching corals (figure 4).

**Figure 5.**
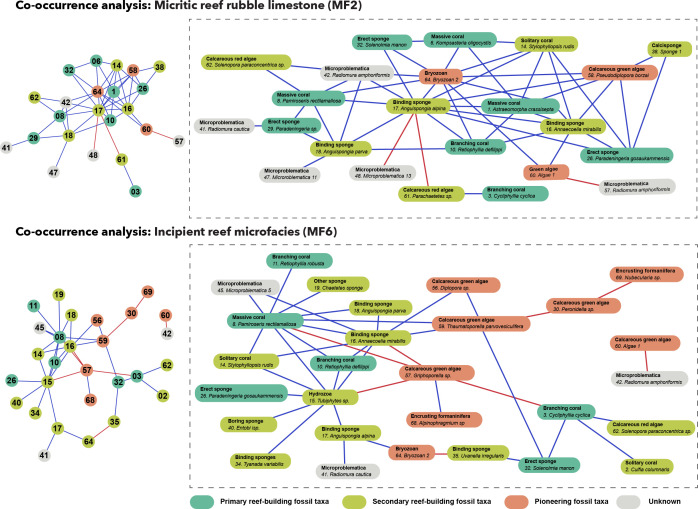
Co-occurrence network. The upper panel displays a network illustrating the non-random co-occurrences among fossil taxa within the micritic reef rubble limestone microfacies (MF2). The lower panel displays a network illustrating the co-occurrences among fossil taxa within the incipient reef microfacies (MF6). Left: original co-occurrence network. Within the network each circle represents a distinct fossil species, with the connecting lines indicating their co-occurrences. Blue lines represent positive co-occurrences, whereas red lines represent negative co-occurrences. Right: a reconstruction of the non-random co-occurrences from the original network. Each node is labelled with its species name and broad taxonomic classification.

Of the non-random associations observed in the co-occurrence analysis for MF2, 41 were positive and 3 were negative (figure 5). Similar to the Spearman correlation analyses, binding inozoan calcisponges show many positive relationships with several primary frame-building organisms as well as pioneering taxa, suggesting that these taxa coexist more frequently than random chance would predict. However, unlike the results from the Spearman correlation analysis, a positive relationship is observed between calcareous red algae and branching corals. The co-occurrence analysis reveals that the binding inozoan calcisponge *Anguispongia alpina* and bryozoans have the most positive co-occurrences within the MF2 microfacies.

Of the non-random associations observed in MF6, 27 were positive and 8 were negative (figure 5). Similar to the results of the Spearman correlation analysis, inozoan calcisponges show a number of positive relationships with secondary frame-building taxa. Additionally, calcareous red algae positively co-occur with a branching coral, *Cycliphyllia cyclica*. The co-occurrence analysis reveals that the hydrozoan *Tubiphytes* sp. and the massive coral *Pamiroseris rectilamallosa* have the most positive co-occurrences within this microfacies.

## 4. Discussion

### (a) Ancient rubble environments

Our study identifies four distinct microfacies within the Late Triassic Dachstein platform, each representing a specific stage in the reef regeneration process following storm events. Spatially, the arrangement of different microfacies illustrates material deposited on and around *in situ* patch reefs after a disturbance event. Temporally, each microfacies corresponds to a particular stage in reef regeneration, characterized by the consolidation of rubble and the progressive increase in structural complexity driven by frame-building organisms. Together, these microfacies capture the structural and ecological development within the regenerating reef system, providing insights into the dynamic processes of reef recovery (figure 2). Although it is not possible to achieve the temporal resolution needed to determine the timing of progression from initial reef rubble formation (MF1) to incipient reef development (MF6) in a way that aligns with modern restoration timescales, our study provides important insights into the types and functional roles of organisms at each stage of reef regeneration.

Reef rubble (MF1) consists of storm-generated material consolidated mainly through abiotic cementation, with no indication of reintegration into the reef framework, as evidenced by the absence of *in situ* fossil taxa, indicating no further reef development. The micritic reef rubble limestone (MF2) represents post-storm settlement deposits acting as the nucleation site for reef development. It is composed of both *in situ* and bioclastic material, exhibiting autochthonous and parautochthonous textures. MF2 is interpreted as representing the initial consolidation stage of reef regeneration, evidenced by early lithification (e.g. hardgrounds) and the overgrowth of pioneering organisms, such as calcareous macroalgae, bryozoans, foraminifera and microbial fabrics.

The incipient reefs (MF6) represent a later stage of rubble consolidation and reef regeneration, characterized by an increase in both secondary reef constituents (e.g. calcareous red algae, microproblematica and microbial fabrics) and primary reef-builders (e.g. calcisponges and scleractinian corals). This microfacies shows evidence of substrate consolidation through intra-interparticle cement, hardgrounds, microbial fabrics and binding calcareous fossil taxa (figure 3). Low-growing erect calcisponges and branching scleractinian corals formed small (2–10 m) reef frameworks (figure 2). Due to their morphology, these reef-building organisms were susceptible to physical damage, particularly in mixed energy environments periodically disturbed by high-intensity hydrodynamic events [25–27]. This accounts for the large amounts of reef rubble observed at the selected field outcrops.

Reef regeneration in modern rubble environments involves a two-phase process: preliminary stabilization of rubble followed by consolidation through rigid binding and lithification [1,8,9]. This study reveals that sessile encrusting calcareous organisms, including calcisponges, bryozoans and calcareous macroalgae, played a critical role in the early stages of rubble consolidation represented by the micritic reef rubble limestone microfacies (figure 3). Binding forms are significantly associated with primary frame-building organisms (e.g. massive corals and erect calcisponges) and pioneering organisms (e.g. bryozoans and encrusting foraminifera) (figures 4 and 5).

Inozoan calcisponges proved to be the most effective taxa for occupying and consolidating rubble beds during the early stages of reef regeneration. Their binding capabilities played a crucial role in consolidating the substrate, potentially facilitating the settlement of primary frame-building organisms, and thereby promoting overall reef development. The observed negative correlation and co-occurrence between binding calcisponges and calcareous red algae may suggest a competitive relationship. In modern rubble environments, both calcareous red algae and sponges are known to readily inhabit and stabilize rubble [8,9,28]. Calcareous red algae typically colonize the upper surfaces of rubble, while sponges that do not rely on light commonly occupy crevices, undersides and deeper areas within the rubble bed [3]. However, living calcisponge species are now mostly restricted to cryptic niches or deeper forereef areas [29–31], making it challenging to directly infer their ecological relationships with red algae in the Triassic. Nevertheless, in thin sections, these fossil taxa are found in similar materials and encrusting comparable substrates, indicating that they likely shared similar environments. This negative association may thus reflect competition for space within these ancient reef ecosystems.

In later stages of rubble consolidation and reef regeneration, represented by the incipient reef microfacies, other sessile-encrusting calcareous organisms take precedence. For example, sphinctozoan calcisponges are more abundant than inozoan calcisponges (figure 3). Additionally, calcareous red algae, which did not show positive relationships in the early stages of reef regeneration, now display positive correlations and co-occurrences with branching corals in the later stages (figures 4 and 5). This suggests that binding inozoan calcisponges may have been more successful in facilitating the settlement of primary frame-building taxa during the early stages of reef regeneration, while calcareous red algae may have played a more prominent role in the later stages along with associated biofilms. Additionally, the diversity and abundance of microbial fabrics (figure 1) indicate the presence of a diverse microbial community. Microbially driven lithification—where microbial biofilms promote cement precipitation—can consolidate loose rubble, aiding in the stabilization and formation of reef structures over geological time [1,10,32,33].

Overall, the early stages of reef regeneration were characterized by higher relative abundances and more positive ecological relationships involving pioneering organisms (e.g. bryozoans, encrusting foraminifera and calcareous macroalgae) alongside secondary frame-builders capable of binding multiple rubble pieces together (e.g. binding inozoan sponges). Binding inozoan calcisponges may have also facilitated the settlement of primary frame-building organisms (i.e. branching corals as well as erect calcisponges). However, the later stages of reef regeneration exhibited higher abundances and more positive ecological relationships involving secondary frame-building organisms that helped fortify the bases of primary frame-building organisms.

### (b) Triassic rubble in a modern context

Many modern coral reef restoration efforts that focus on stabilizing substrates use artificial structures like concrete blocks, domes or mats [34]. However, natural materials often yield higher coral recruitment and diversity, and successional processes that stabilize substrates could further enhance restoration outcomes, yet these approaches are frequently overlooked [35–39].

Erect reef sponges provide a promising natural alternative for stabilizing rubble, creating a foundation for carbonate-secreting organisms to bind it securely [40,41]. On a shallow Panamanian reef (2−3 m), Wulff [41] found that rubble piles with added sponge fragments maintained an average height of 8.6 cm after 2 weeks, compared to 4.5 cm for rubble-only piles, which were eventually scattered by wave action and bioturbation. In Curacao, Biggs [40] observed that sponge fragments attached to rubble within 2−4 days and stabilized 90% of rubble pieces over 24 months. Similarly, Kenyon *et al.* [2] found that rubble piles in the Maldives developed binds primarily from sponges within six months. Cryptic sponges, which stabilize rubble from within, further enhanced binding by adhering to over 50% of rubble piles within one month and achieving 73% stabilization after five months [41].

Beyond rapid stabilization, erect sponges attract benthic invertebrate larvae [42], and their growth form allows coral settlement with minimal interference. Branching sponges propagate asexually [41,43] and can reattach quickly to substrates using any part of their tissue [44], making them well suited for occupying and stabilizing coral rubble.

Experimental results demonstrate multiple advantages of sponge-seeding—the strategic placement of sponge fragments on rubble to stabilize loose substrates [40]. Sponges provide stable, natural substrates that enhance coral recruitment, support coral diversity and offer a sustainable, cost-effective option for developing regions [40]. Biggs [40] showed that coral larvae prefer rubble stabilized by natural agents, with the highest recruitment on sponge-seeded rubble piles. As sponge seeding is a relatively new technique, additional research is needed to evaluate its effectiveness and ecological impact.

In modern reefs, most sponges provide only temporary stabilization, as they lack rigid calcareous skeletons. However, the calcisponges in our samples, which did produce rigid structures, likely contributed to long-term rubble consolidation. Our analysis revealed diverse calcisponges acting as critical binders of reef rubble and highlighted a positive correlation between scleractinian corals and certain calcisponges, suggesting a potential role of calcisponges in facilitating coral recovery and reef regeneration (figures 4 and 5).

Our findings underscore the critical role of sponges in natural reef regeneration. This study demonstrates how specific sponges contribute to both immediate stabilization and longer-term consolidation of reef structures. While sponges play a pivotal role in rubble stabilization, it is important to note that most modern reef sponges are soft-bodied and do not provide the rigid framework needed for structural consolidation. Consequently, sponge seeding should be considered a valuable strategy for stabilizing loose rubble in the short term. For long-term consolidation and the development of a durable reef framework, it is equally important to support the recruitment of calcifying, laterally encrusting organisms such as calcareous red algae and bryozoans, which provide the rigid structure essential for sustained reef stability. Our findings reveal calcareous red algae-bound rubble in both MF6 and MF2, often in close association with branching scleractinian corals, suggesting their role in enhancing coral recovery after disturbances (figures 4 and 5). Together, these natural agents offer a complementary approach that can enhance both the immediate and enduring success of restoration efforts. This research supports the further exploration and refinement of sponge-mediated coral restoration methods in hopes of leading to more resilient reef ecosystems capable of withstanding future disturbances.

Notably, despite the presence of key natural consolidators, these Triassic reefs exhibited limited structural complexity, rarely exceeding 10 m in diameter and consistently remaining under 2 m in height. Many reefs within a single transect did not continue into the overlying transect, often becoming buried by rubble. This indicates that even with natural stabilizers, achieving substantial structural complexity may not always be possible. Realistic expectations for reef restoration must consider factors such as storm frequency and intensity, as well as the morphology and physiology of primary frame-building organisms, which influence their capacity to withstand physical stress and recover [26]. With storm intensity projected to increase, particularly in the Atlantic and West Pacific [45,46], the frequency of intensified storms may exceed coral recovery timeframes [47,48]. These factors underscore the need for adaptive strategies in reef restoration that account for the dynamic and intensifying challenges posed by climate change.

## 5. Conclusions

Our study reveals that the processes of reef rubble consolidation and regeneration observed on the Late Triassic Dachstein platform mirror those in modern coral reefs, indicating an enduring pattern in reef development. The distinct microfacies we identified reflect successive stages of reef recovery, starting with the initial consolidation of rubble by pioneering organisms and secondary frame-builders, progressing to advanced consolidation by calcareous red algae and calcisponges, which create favourable conditions for coral settlement and structural complexity. This enduring ecological strategy emphasizes the critical importance of these natural processes for contemporary reef conservation and restoration, especially given the rise in climate-related disturbances.

Our findings contribute insights into the dynamics of long-term reef regeneration. By pinpointing taxonomic groups that have historically played essential roles in rubble consolidation, we can prioritize these organisms in modern rubble habitat studies and restoration efforts. Our study encourages further research into sponge-seeding as a promising approach to enhance coral recruitment, support biodiversity and offer a cost-effective restoration solution. Yet, long-lasting reef stability depends on the recruitment of calcifying organisms like calcareous red algae and bryozoans.

Triassic reefs, despite the presence of these stabilizers, exhibited limited structural complexity and were frequently disrupted by high-energy events, highlighting the need for realistic restoration targets that consider environmental variability. With climate change driving an increase in storm intensity, restoration approaches must adapt to support reef systems resilient to ongoing and future disturbances.

## Data Availability

The code and data associated with this study are freely available on the Open Science Framework (OSF) at https://doi.org/10.17605/OSF.IO/GBECX [49] under permissive licences: the data are licensed under CC-BY 4.0, and the code under Apache 2.0. Supplementary material is available online [50].
